# The Penalty of Neglecting Veterinary Medicine

**Published:** 1902-08

**Authors:** 


### EDITORIALS.

### THE PENALTY OF NEGLECTING VETERINARY MEDICINE.

We have often urged upon our readers the precept that it was not wise for practitioners of medicine to fail to inform themselves to some extent in comparative pathology. What comes of such neglect? Ignorance of the connection between disease in the lower animals and disease in man, ignorance even of the existence among human beings of certain malignant diseases of domestic animals. The veterinarians properly insist upon the importance of their work from the point of view of the public health, but seldom so cogently as was recently done by W. H. Dalrymple, M.R.C.V.S., of the Louisiana State University, in a paper entitled "The Value of Co-operation in the Sanitary Control of Our Periodic Epizootics of Anthrax," read before the Louisiana State Medical Society in June, and published in the August number of the *New Orleans Medical and Surgical Journal.*

Those of us—and we are not a few—who have seen occasional cases of malignant pustule in the human subject do not need to be told that Surgeon-General Sternberg was in error when he stated in his *Text-book of Bacteriology* that anthrax did not prevail in the United States, unless, indeed, he used the word "prevail" in a sense that would suggest an ever-present pestilence; but few of us probably are aware of the amount of devastation wrought by the disease in Louisiana and Mississippi. A striking picture of the facts is given by Professor Dalrymple, and his recommendations for restricting outbreaks are such as must commend themselves to those who reflect upon the situation. But it is not to these features of his very interesting communication that we shall now direct attention, but to the eloquent plea that he makes for comparative pathology as a subject of thought with the medical profession in general.

He says he knows of country practitioners who have turned their veterinary knowledge to account in times of anthrax epizootics by informing the people of their danger and inculcating such sanitary precautions as the complete destruction of the carcasses of animals that have succumbed to the disease and the practice of thorough disinfection, and the results have been brilliant. "But," he adds, "I have heard of others who, on being asked for information, because the victim of anthrax happened to be a mule or a cow, explained with an air of wounded dignity, 'I'm no mule or cow doctor, and don't know anything about it.'" The dignity that needs to be so safeguarded must, we should say, be made of very unsubstantial stuff. The result of such a reply, says the author, has often been that some illiterate person, without any sanitary knowledge whatever, has been called in, and the contagion been permitted to spread broadcast. It does not, he aptly says, indicate the true pathologist to disclaim all interest in the disease of the lower animals, for he "looks upon disease as such, and does not consider the subject that accidentally has become the victim of it." And he is quite justified in deprecating forgetfulness of the fact that "the magnificent strides medical science has taken and the exalted pinnacle to which it has attained in recent years" have been largely owing to the efforts of veterinarians.—*New York Medical Journal,* August 9, 1902.

